# Characterization and Prediction of Physical Properties of Luanta Fir Wood with Vacuum Hydrothermal Treatment

**DOI:** 10.3390/polym14204374

**Published:** 2022-10-17

**Authors:** Ming-Chi Hsieh, Ke-Chang Hung, Jin-Wei Xu, Wen-Shao Chang, Jyh-Horng Wu

**Affiliations:** 1 Department of Forestry, National Chung Hsing University, Taichung 40227, Taiwan; 2 Department of Wood Based Materials and Design, National Chiayi University, Chiayi 600355, Taiwan; 3 School of Architecture, University of Sheffield, Sheffield S10 2TN, UK

**Keywords:** *Cunninghamia konishii*, near infrared spectroscopy, nondestructive spectroscopy technique, prediction model, physical property, heat treatment

## Abstract

This study used the luanta fir (*Cunninghamia konishii* Hayata) wood, one of the most used wood construction and building materials in Taiwan, as specimens to examine the impact of different conditions of vacuum hydrothermal (VH) treatment on the physical properties of this wood. A prediction model for these properties was created using a nondestructive spectroscopy technique. The test results revealed that the mass loss, moisture exclusion efficiency, anti-swelling efficiency, color difference, and surface contact angle of the VH-treated wood all increased under increasing heat treatment temperature and time. Moreover, the use of near-infrared (NIR) spectroscopy in creating the prediction model for the physical properties of the VH-treated luanta fir wood revealed that the ratios of performance to deviation (RPD) for mass loss, equilibrium moisture content, and color difference were all above 2.5, indicating a high prediction accuracy. These results suggested that an NIR spectrometer can serve as a useful instrument for the accurate prediction of the physical properties or for controlling the quality of VH-treated wood.

## 1. Introduction

Wood is a natural and renewable biomaterial commonly used for its high specific strength, machinability, and moisture absorption and desorption. However, when used outdoors, wood as an anisotropic biomaterial can be easily deformed, warped, and cracked under the effect of moisture absorption and desorption and can be damaged through sun exposure, rainfall, and microorganisms [1,2]. According to research, heat treatment can effectively improve wood properties including its dimensional stability, durability, and decay resistance, and constitutes an environment-friendly wood modification method [3,4,5]. Among the numerous heat treatments, closed hydrothermal treatment not only increases the moisture content and reduces cracking in wood, but also enhances the mobility of polymer chains and reduces the activation energy of polymers in noncrystalline regions, thereby raising the degradation rate and further reducing energy consumption during heat treatment [6,7]. Vacuuming before heating removes the air in the processing tank and wood, which ensures a low-oxygen environment for heat treatment and lowers the boiling point of the water. Consequently, liquid water is vaporized in a shorter time, enabling the inside of the wood cells to be filled with vapor at a lower temperature, which effectively reduces wood cracking [5]. The wood of luanta fir, a tree species endemic to Taiwan, is advantageous for its straight texture and lightness, and is one of the most used wood construction and building materials in Taiwan [8]. Therefore, through applying vacuum hydrothermal (VH) treatment, this study explored the impact of different conditions of heat treatment on the physical properties of luanta fir wood.

Generally, the mass loss (ML) of heat-treated wood increases with the increased time, temperature, and pressure of heat treatment [9,10]. ML mainly results from water evaporation and hemicellulose degradation, which generates degradation products such as formic acid and acetic acid, degrading the noncrystalline regions of cellulose. Although the phenomenon effectively decreases the number of the accessible hydroxyl groups in wood and increases its dimensional stability and durability, its mechanical strength is reduced [5]. This illustrates the effects of the numerous chemical reactions occurring during heat treatment on the properties of heat-treated wood. Therefore, the analysis and evaluation of the chemical composition of heat-treated wood enable its effective utilization. Specifically, near-infrared spectroscopy (NIRS) is a promising analytical technique. As a nondestructive technique for testing, NIRS requires no specific pretreatment, shortens testing time, and saves on testing costs compared with traditional techniques, and enables the precise prediction of wood components. Accordingly, numerous studies have used NIRS in the regression analysis of wood properties and have developed relevant models for predicting wood properties [11,12,13]. This study explored the impact of different VH conditions on the physical properties of luanta fir wood and applied the nondestructive NIRS technique to create prediction models for the relevant properties of VH-treated wood.

## 2. Materials and Methods

### 2.1. Materials

The luanta fir (*Cunninghamia konishii* Hayata) wood used for testing was purchased from the Jang Chang Lumber Industry Co., Ltd. (Hsinchu, Taiwan). The acquired material was cut into specimens with a length (longitudinal direction), width (tangential direction), and thickness (radial direction) of 600, 138, and 26 mm, respectively. Before VH treatment, all specimens were conditioned at 20 °C and 65% relative humidity (RH) for at least 2 weeks, and the specimens that demonstrated a modulus of elasticity (MOE) between 6.5 and 5.0 GPa under a load of 10 N were selected for the subsequent tests to reduce the influence of individual variations on the specimen properties.

### 2.2. Vacuum Hydrothermal Treatment of Luanta Fir Wood

The moisture-conditioned wood was placed in a semi-industrial reactor with temperature and pressure controls (San Neng, Chiayi, Taiwan). The wood and a proper amount of water were placed in the tank without contact with each other, and a vacuum pump was used to lower the internal pressure to <23 kPa. Subsequently, heating was conducted at a heating rate of 3 °C/min to achieve temperatures of 160, 180, 200, 220, and 240 °C, followed by heat treatments lasting 4, 8, and 16 h (these temperature ranges and durations are frequently used for wood heat treatment). The specimens were then cooled to room temperature. Specimens then underwent moisture conditioning at 20 °C with 65% RH in preparation for subsequent experiments, and 15 replicate specimens were tested for each group.

### 2.3. Measurement of Mass Loss

Carbohydrate degradation products formed during closed hydrothermal treatment are generally stratified in wood, and such degradation products cannot be removed in a 105 °C oven, leading to the underestimation of the ML of heat-treated wood [9,14]. Therefore, in reference to the testing technique of Zeniya et al. [15], the present test involved subjecting oven-dried specimens with dimensions of 20 mm × 20 mm × 20 mm to hot water extraction. The products were dried to constant weight in an oven set at 105 °C, and their weights were recorded to calculate the water-soluble components (WSCs) per 100 g of wood. The ML of the specimens was calculated according to the following equations:

WSC (g/100 g dw) = [(*m*_d,h_ − *m*_w,h_)/*m*_d,h_] × 100 (1)

*m*_c_ (g) = *m*_d_ − [(*m*_d_ × *WSC*)/100] (2)

ML (%) = [(*m*_c,u_ − *m*_c,h_)/*m*_c,u_] × 100 (3)

where *m*_d,h_ and *m*_w,h_, respectively, represent the VH-treated wood’s oven-dry mass (g) before and after hot water extraction, *m*_c_ represents each specimen’s calibrated oven-dry mass (g), *m*_d_ represents the specimen’s oven-dry mass (g), and *m*_c,u_ and *m*_c,h_ represent the calibrated oven-dry masses of the untreated and VH-treated wood, respectively. Sample mass was determined to a precision of 0.01 g.

### 2.4. Determination of Physical Properties

To determine the physical properties of untreated and VH-treated luanta fir wood, several determinations, including air-dry density, equilibrium moisture content (EMC), moisture excluding efficiency (MEE), water absorption, volumetric swelling coefficient (S), and anti-swelling efficiency (ASE), were conducted. In brief, the air-dry density and EMC of cubic specimens with dimensions of 20 mm × 20 mm × 20 mm were measured according to the Chinese National Standards CNS 451 [16] and CNS 452 [17], respectively. The moisture exclusion efficiency (MEE) was calculated by Equation (4):

MEE (%) = [(EMC_u_ − EMC_h_)/EMC_u_] × 100 (4)

where EMC_u_ and EMC_h_ denote the EMC (%) of the untreated wood and VH-treated wood, respectively. The water absorption and S were measured according to CNS 14927 [18]. The S and ASE were calculated by Equations (5) and (6):

S (%) = [(*L*_t1_ × *L*_r1_ − *L*_t0_ × *L*_r0_)/(*L*_t0_ × *L*_r0_)] × 100 (5)

ASE (%) = [(S_u_ − S_h_)/S_u_] × 100 (6)

where *L*_t1_ and *L*_r1_ are the specimen’s tangential and radial dimensions (mm) over the fiber saturation point, *L*_t0_ and *L*_r0_ are its tangential and radial dimensions (mm) in an oven-dry condition, and S_u_ and S_h_ represent the S (%) of the untreated and VH-treated wood, respectively. The dimensions of the specimens were determined to a precision of 0.01 mm.

### 2.5. Measurement of Surface Colour

The color of untreated and VH-treated luanta fir wood was measured by a color and color difference meter (CM-3600d, Minolta, Tokyo, Japan) under a D_65_ light source with a test window diameter of 30 mm. The color parameters L*, a*, and b* of all specimens were obtained directly from the colorimeter, and the value was determined to a precision of 0.01. Based on the CIE L*a*b* color system, L* is the value on the white/black axis, a* is the value on the red/green axis, b* is the value on the yellow/blue axis, and the ΔE* value is the color difference (Equation (7)).

ΔE* = [(ΔL*)^2^ + (Δa*)^2^ + (Δb*)^2^]^1/2^ (7)

### 2.6. Measurement of Surface Contact Angle

A Kyowa CA contact angle meter (Kagawa, Japan) was used to measure the contact angles of the water droplets on the surfaces of the specimens. Distilled water droplets were applied to measure the contact angles within 15 s of the treatments. The contact angle of the specimens was determined to a precision of 2°.

### 2.7. NIR Spectral Measurements

A PerkinElmer Spectrum Two N (Buckinghamshire, UK) NIR spectrometer was used to determine functional group changes on the surfaces of the untreated and VH-treated specimens. The spectra were collected by co-adding 32 scans at a resolution of 4 cm^−1^ in the range from 10,000 to 4000 cm^−1^.

### 2.8. Establishing Prediction Model for Wood Properties

**Figure 1:**
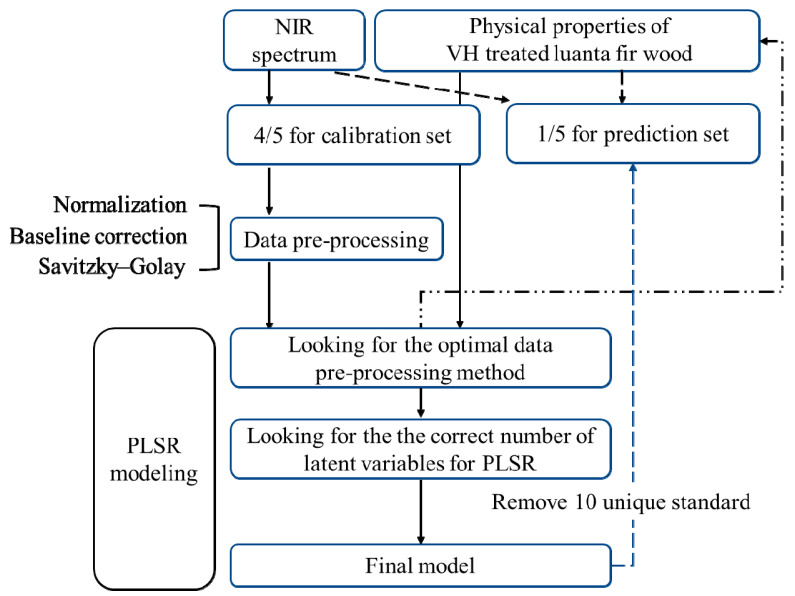
Schematic diagram of a prediction model for wood physical properties based on NIRS.

The PerkinElmer Spectrum 10 software (Buckinghamshire, UK) with spectrum quant advanced algorithms pack (10.6.2.1159 ver.) was used to analyze the physical properties and NIR spectra of the VH-treated specimens and to construct the property prediction models. According to the processing steps presented in Figure 1, for each property, 240 data entries were obtained from the one untreated set and 15 VH-treated sets, each of which was tested 15 times, except for the ML and color difference processing, which involved no untreated set. The data of 80% of these entries formed the calibration set, and the data of 20% of these entries formed the prediction sets. Prior to model creation, the spectra were subjected to preprocessing techniques including normalization (i.e., multiplicative signal correction [MSC]) and the standard normal variate [SNV] method), baseline correction, derivative, and Savitzky–Galay (SG) filtering. For the method combining first derivative and SG filtering, the number of points for smoothing was set as 5, and for the method combining second derivative and SG filtering, the number of points for smoothing was set as 49. Subsequently, the number of latent variables (LVs) was set to 10, partial least squares regression (PLSR) was used to create the model, and the cross-validation (leave-one-out technique) was used in all cases for calibration model evaluation. The optimal preprocessing condition was evaluated by the coefficient of determination (*R*^2^), root mean square error (RMSE) of calibration (RMSEC), and RMSE of cross-validation (RMSECV). The 10 data entries failing to correspond to this model were removed. The final prediction model was created using identical pretreatment conditions and LVs. Finally, verification was conducted through substituting the prediction sets in this model to calculate the RMSE of prediction (RMSEP) and ratio of performance to deviation (RPD) using the actual data in the prediction sets, thereby enabling evaluation of the model accuracy.

### 2.9. Statistical Analysis

All results are expressed as the mean ± standard deviation (SD). The significance of differences was calculated using Scheffe’s test or Student’s *t*-test, and *p* values < 0.05 were considered to be significant.

## 3. Results and Discussion

### 3.1. ML and Air-Dry Density

The properties of VH-treated wood are affected by the level of thermal degradation. Accordingly, testing was conducted to assess the effect of different VH treatment conditions on the level of thermal degradation in wood through an analysis of the ML of VH-treated wood. The findings presented in Table 1 revealed that the ML of the VH-treated wood increased under increasing treatment temperatures and duration times, largely because of the vaporization of hygroscopic water and extracted components in the early stage of heat treatment [19,20]. However, when the temperature and time of the heat treatment increased, degradation of the hemicellulose in the wood occurred, generating acid degradation products such as formic acid and acetic acid, and thus prompting polysaccharide and lignin degradation. The phenomenon was enhanced under an increasing treatment temperature and duration time. In terms of ML, significant differences were observed between the woods treated under distinct conditions, specifically, at 240 °C for 8 h (18.0%) and at 240 °C for 16 h (31.2%), and the other sets, indicating that cellulose may also degrade considerably during heat treatment at high temperatures and for long durations; hence, a significant increase in the ML of the luanta fir wood was observed. Moreover, the findings relating to air-dry density presented in Table 1 indicate that the air-dry density of the untreated luanta fir wood was 416 kg/m^3^. Among the VH-treated specimens, no significant difference was observed among the air-dry density values (389–414 kg/m^3^) of the woods treated under different conditions, except for those treated at 240 °C for 8 and 16 h. However, an evaluation based on Student’s *t* test revealed significant differences between the air-dry density of the untreated set and the air-dry densities (354–374 kg/m^3^) of the woods subjected to VH treatments under the following conditions: 160 °C for 16 h, 180 °C for 16 h, 200 °C for 16 h, 220 °C for 8 h, and 220 °C for 16 h. Moreover, significant differences (*p* < 0.001) were observed between the air-dry densities of the woods prepared using VH treatments at high temperatures for long durations (i.e., at 240 °C for 8–16 h) and the air-dry density of the untreated set, indicating that the specimens’ air-dry densities decreased under increasing temperatures and times during heat treatment.

### 3.2. Hygroscopicity and Water Absorption

To assess the effect of different conditions in VH treatment on the hygroscopicity of wood, wood was conditioned to constant weight in an environment at 20 °C and 65% RH, and its EMC and MEE were calculated. The test results reported in Table 1 reveal that the EMC of the untreated wood was 8.3%; among the treated wood, the EMC value decreased under increasing treatment temperature and time. The lowest EMC (3.7%) was observed in the wood treated at 240 °C for 16 h, and its MEE reached 56%, verifying the aforementioned inference relating to air-dry density. Additionally, the Student’s *t* test results revealed a significant difference in EMC between the untreated wood and the wood treated at 160 °C for 4 h (EMC = 7.9%; MEE = 5%), indicating that VH treatment at lower temperatures and for short durations effectively decreased wood hygroscopicity. Compared to the results reported by Altgen and Militz [21], the heat-treated European beech with similar ML exhibited higher EMC than VH-treated luanta fir wood.

Moreover, to evaluate the impact of different VH treatment conditions on the water absorption and dimensional stability of wood, wood was immersed in 20 °C water to calculate the S and ASE values. As shown in Table 1, the S value of the untreated luanta fir wood was 13%, and, after VH treatment, the S values of the specimen sets decreased under increasing treatment temperature and duration time. Moreover, under VH treatment at 160 °C for 4 h, significant differences were observed in the S value between the treated and untreated specimens. When the treatment conditions increased to 160 °C and 8 h, major significant differences were observed between the S values of the treated and untreated wood, indicating that VH treatment at 160 °C effectively improved the dimensional stability of luanta fir wood. Additionally, except for the wood treated at 240 °C for 16 h, the ASE values of the specimen sets increased under increasing treatment temperature and duration time, with the highest S value (63%) observed in the wood treated at 240 °C for 8 h. A summary of the aforementioned test results indicates that VH treatment can effectively decrease the hygroscopicity and volumetric swelling coefficient of wood. The phenomenon may be attributed to the following three reasons [22,23,24]. Firstly, during VH treatment, hydronium ions (H_3_O^+^) accelerate the degradation reaction of polysaccharides, decreasing the accessible hydroxyl groups in wood and reducing the absorption positions of water molecules, hence decreasing the hygroscopicity of wood. Secondly, the acid degradation products generated during hemicellulose degradation resulted in the degradation of cellulose in noncrystalline regions, increasing the relative crystallinity of wood and preventing moisture from entering. Thirdly, rearrangement and crosslinking reactions are generated from the thermal degradation products of lignin and polysaccharides. The number of carbonyl groups in wood decrease, and the degradation of polysaccharides generate more hydrophobic furfural and its derivatives, which decrease the hydrophilicity of wood.

### 3.3. Surface Contact Angle

According to Kocaefe et al. [4], the surface hydrophobicity of wood increases after heat treatment, thus altering the surface contact angle. As shown in Table 1, the surface contact angle of the untreated luanta fir wood was 60°, and that of the VH-treated wood generally increased under increasing treatment temperature and time. The greatest surface contact angle (104°) was observed in the wood treated at 200 °C for 16 h, which was approximately 73% higher than that of untreated wood. No significant difference was observed in the values of surface contact angles under increasing treatment temperature and time. This result was mainly attributed to the degradation and condensation of polysaccharides during heat treatment, which produced furfural molecules and thus reduced hydrophilicity. With the extractives migrating to the wood surface, the polarity of the wood surface decreased and its hydrophobicity increased [25,26]. Moreover, these findings correspond with the results of hygroscopicity and water absorption.

### 3.4. Surface Colour Change

**Figure 2:**
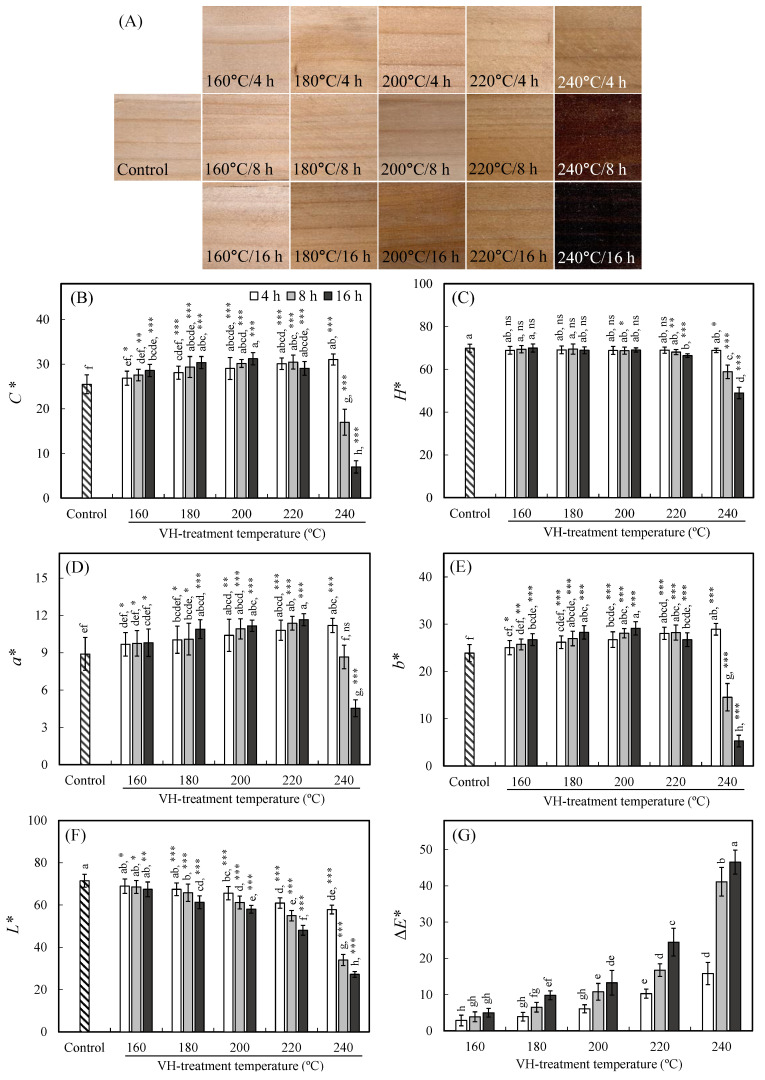
Surface images (**A**), C* (**B**), H* (**C**), a* (**D**), b* (**E**), L* (**F**), and ΔE* (**G**) of untreated and VH-treated luanta fir wood. Values are mean ± SD (*n* = 15). Bars with different letters indicate significant differences (*p* < 0.05). ns: non-significant; *: *p* < 0.05; **: *p* < 0.01; ***: *p* < 0.001 (one-tailed test) compared with control.

During heat treatment, the main components of wood degrade, thus altering the color of the wood. To further assess the impact of different VH treatment conditions on wood surface color change, images of the specimen surfaces and color parameters were analyzed. As illustrated in Figure 2A, the wood surface color deepened under increasing treatment temperature and time. This phenomenon was mainly attributed to the degradation products containing conjugated double bonds or quinones generated in wood during heat treatment [27]. However, when treatment temperature and time were respectively increased to 240 °C and 8–16 h, the specimen surfaces changed to a dark brown color. Figure 2B–G depicts the C*, H*, a*, b*, L*, and ΔE* values of the specimen sets; C* (Chroma) is the purity of a color, and H* (Hue) is the attribute of a visible light, due to which it is differentiated from or similar to the primary colors. Based on the chromas of the specimens presented in Figure 2B, the C* value of the untreated luanta fir wood was 25.5. After VH treatment, this value had increased under increasing temperature and duration time, and the highest C* value (31.2) was observed in the specimen treated at 200 °C for 16 h. However, when the VH conditions were changed to 240 °C for 8 and 16 h, the C* values of the specimens significantly decreased to 17 and 7, respectively. By contrast, the H* value of the untreated luanta fir wood was 69.9 (Figure 2C). After VH treatment, the H* values of the sets of the VH-treated wood ranged from 68.0 to 69.4, with no significant difference observed between these values, except for a markedly significant difference observed between the untreated set and the sets treated at high temperatures (>220 °C) and for long durations (>8 h); their H* values were 66.4 (220 °C/16 h), 58.8 (240 °C/8 h), and 48.9 (240 °C/16 h).

As illustrated in Figure 2D,E, the a* and b* values of the untreated luanta fir wood were 8.9 and 23.9, respectively. The variations in the trends of a* and b* values of the VH-treated specimens were similar. When the heat treatment temperature was lower than 240 °C, the a* and b* values both increased slightly, with the greatest a* value (11.7) and b* value (28.9) observed in the specimens treated at 220 °C for 16 h and at 240 °C for 4 h, respectively. However, evident decreases were observed in the a* and b* values of the specimens when the treatment conditions changed to 240 °C/8 h (a* value = 8.7; b* value = 14.6) and 240 °C/16 h (a* value = 4.5; b* value = 5.3). Moreover, the L* values of the wood, as shown in Figure 2F, decreased under increasing VH treatment temperature and time, with these values decreasing from 71.5 in the untreated wood to 27.2 in the wood treated at 240 °C for 16 h. By contrast, the ΔE* values of the VH-treated wood increased under increasing treatment temperature and time, and the highest ΔE* value (46.5) was observed in the specimen treated at 240 °C for 16 h (Figure 2G). The possible main reasons for changes in the wood surface color after heat treatment are as follows: (1) colored substances such as furfural, hydroxymethylfurfural, and anhydroglucose are produced from the hemicellulose or cellulose during thermal degradation; (2) cleavage of the *β*–*O*–4 linkage in lignin causes a decrease in the content of methoxy groups and an increase in the content of chromophore containing unsaturated double bonds and benzene-ring structured compounds, thus deepening the color of wood; (3) in VH-treated wood, the relative lignin content increases, thus further increasing the effect of lignin-containing additional chromophores; and (4) the degradation of the extractives and their migration to the wood surface also deepens the color of wood [28,29,30,31,32].

### 3.5. NIRS Analysis

**Figure 3:**
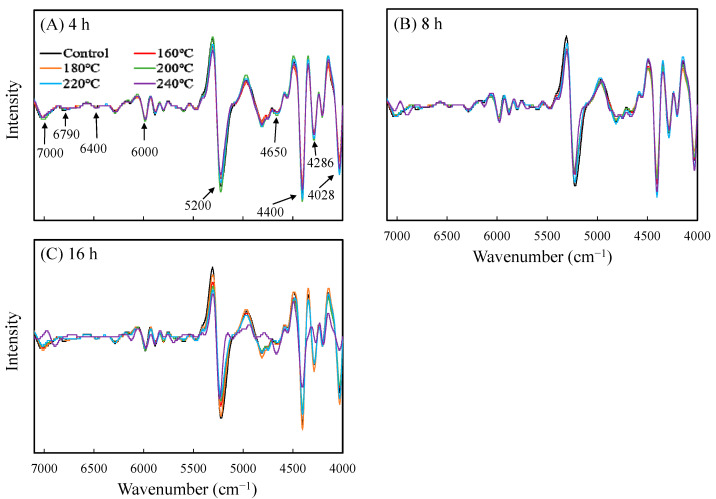
NIR spectra preprocessed by the second derivative combined with SG of luanta fir wood after VH treatment at different temperatures for 4 (**A**), 8 (**B**), and 16 h (**C**).

To understand the changes generated under different VH treatment conditions relative to the chemical structure of luanta fir wood, a test was performed to explore the differences between the specimens subjected to various VH treatments using NIR spectra processed through the second derivative combined with SG filtering. In general, the downward absorption peak of the second derivative spectrum marks the location of the absorption peak of the initial spectrum. Accordingly, in the spectra of the untreated luanta fir wood presented in Figure 3, the following absorption peaks were observed: apparent characteristic absorption peaks of the hydroxyl groups in cellulose in the noncrystalline, hemicrystalline, and crystalline regions at 7000, 6790, and 6400 cm^−1^, respectively; the characteristic absorption peak of the hydroxyl groups in water molecules at 5200 cm^−1^; the characteristic absorption peak of the hemicellulose at 6000 and 4286 cm^−1^; the characteristic absorption peak of the lignin and extract at 4650 cm^−1^; the bending and deformation vibration absorption peak of the C–H functional groups of the cellulose and hemicellulose at 4400 cm^−1^; the stretching vibration absorption peak of the C–H and C–C functional groups at 4189 cm^−1^; and the stretching vibration absorption peak of the C–H and C–C functional groups of the cellulose and lignin at 4028 cm^−1^. In the VH-treated wood, the intensity of the absorption peak of the hydroxyl groups at 7000, 6790, and 5200 cm^−1^ and the intensity of the characteristic absorption peak of the hemicellulose at 6000, 4400, and 4286 cm^−1^ both decreased under increasing treatment temperature and time. The findings indicated that, in the VH-treated wood, degradation to the noncrystalline regions of hemicellulose and cellulose occurred, causing a dehydration reaction. However, in the VH-treated wood, the intensity of the characteristic absorption peak of the lignin at 4650 and 4028 cm^−1^ increased under increasing treatment temperature and time; following VH treatment, the relative lignin content had increased [33,34]. Moreover, as shown in Figure 3B,C, in the wood samples treated at 240 °C for 8 and 16 h, the characteristic absorption peak of the crystalline regions of cellulose at 6400 cm^−1^ was almost invisible, indicating that cellulose had begun to degrade under such conditions.

### 3.6. Establishing the Prediction Model

**Figure 4:**
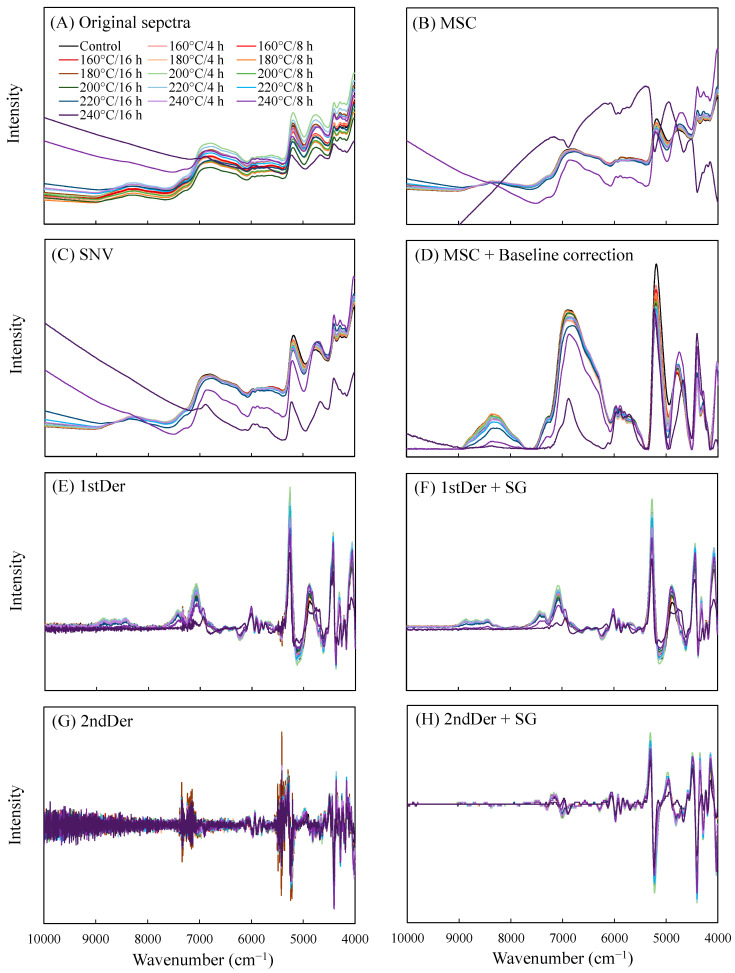
NIR spectra before (**A**) and after preprocessing by MSC (**B**), SNV (**C**), MSC + baseline correction (**D**), first derivative (**E**), first derivative + SG (**F**), second derivative (**G**), and second derivative + SG (**H**).

To evaluate the properties of VH-treated wood while controlling its quality using simple and rapid techniques, PLSR was applied to analyze NIR spectra data as well as the different physical properties of the VH-treated wood, including the air-dry density, ML, EMC, S, contact angle, and ΔE*, thus enabling the constructing of a prediction model for these properties. Each of these properties was assessed using 240 data entries, except for ML and ΔE*, for which 225 data entries each were assessed. The calibration set was formed using 80% of the data, with the remaining 20% used for the prediction sets. The ranges of values for each of these properties are listed in Table 2. Notably, to prevent extrapolation, the ranges of the values in the prediction sets must not exceed those of the data in the calibration set. Moreover, when solid samples are subjected to NIRS analysis, the scattering effect, molecular interactions, changes in the refractive index of light, and specimen size all affect the NIR spectra and lead to noise or baseline shifts. Therefore, prior to creating a model, preprocessing is required for signal enhancement and noise reduction in the spectra. In this study, preprocessing techniques such as MSC, SNV, baseline correction, derivative, and SG filtering were applied and the results were as shown in Figure 4. The process of preprocessing selection is often limited to trial-and-error and is therefore considered somewhat subjective. In this study, to select the optimal pretreatment conditions, all the PLSR-analyzed LVs were set to 10, and the *R*^2^ and RMSEC values for the preprocessed models were calculated. The test results presented in Table 3 revealed that, for all the properties, the highest *R*^2^ value and lowest RMSEP value were observed in the prediction model preprocessed using the second derivative in combination with SG filtering. The test results are similar to those of Yu et al. [35]. A subsequent test was conducted, with preprocessing conducted using the second derivative combined with SG filtering. Moreover, to avoid overfitting and to increase model representativeness (i.e., lower LV and RMSEC values), this test was conducted in reference to the testing technique of Mitic et al. [36], with LVs with the following conditions selected as the optimal conditions: (1) a difference between the RMSEC and RMSECV of less than 20%; and (2) having the minimal RMSEC value. Accordingly, the optimal LVs of air-dry density, ML, EMC, S, contact angle, and ΔE* were 4, 5, 5, 5, 4, and 4, respectively (Table 4).

**Figure 5:**
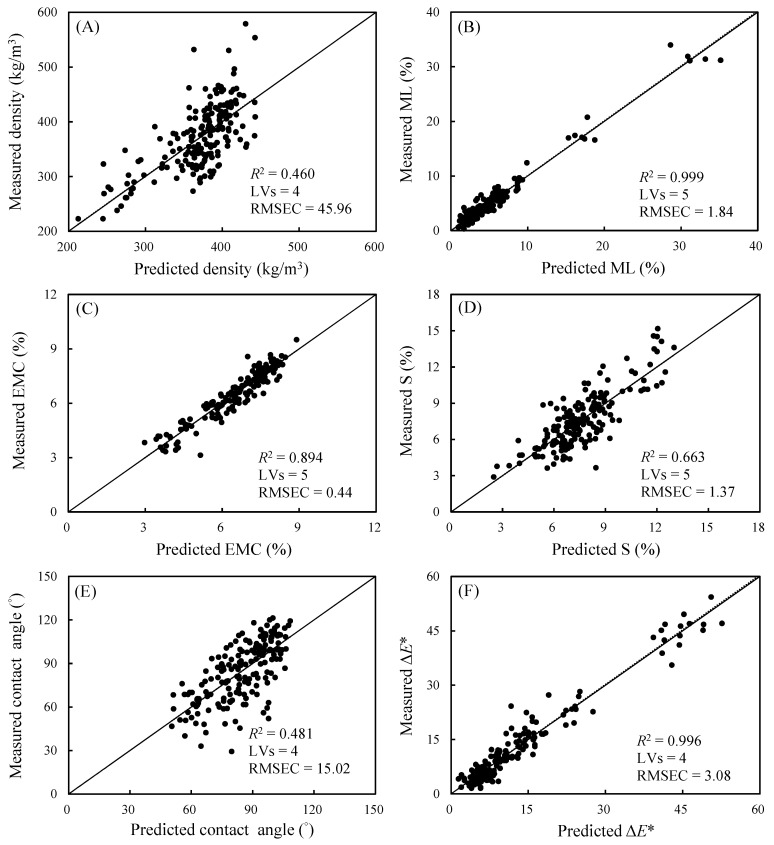
The correlation between predicted and measured values of air-dry density (**A**), ML (**B**), EMC (**C**), S (**D**), contact angle (**E**), and ΔE* (**F**) in calibration data.

Figure 5 present a correlation graph for the predicted and measured values of the specimens’ physical properties obtained through modeling under the aforementioned conditions. Linear regression through the origin for each of the properties revealed *R*^2^ values greater than 0.9 for ML (0.999) and ΔE* (0.996), indicating highly favorable modeling effects. Specifically, *R*^2^ values greater than 0.5 were observed for EMC (0.894) and S (0.663), with the lowest *R*^2^ value observed for air-dry density (0.460). Moreover, lower RMSEC values (<2) in the prediction model were observed for three properties, namely EMC, ML, and S, and their mean values were less than 10 (as detailed in Table 2). The findings indicate that the magnitude of the RMSEC was mainly affected by the range of values for individual properties, and is thus unfit for evaluating the differences in one prediction model in terms of different properties; the RMSEC can thus only be applied to compare different modeling techniques in relation to a single property.

### 3.7. Model Prediction Results

**Figure 6:**
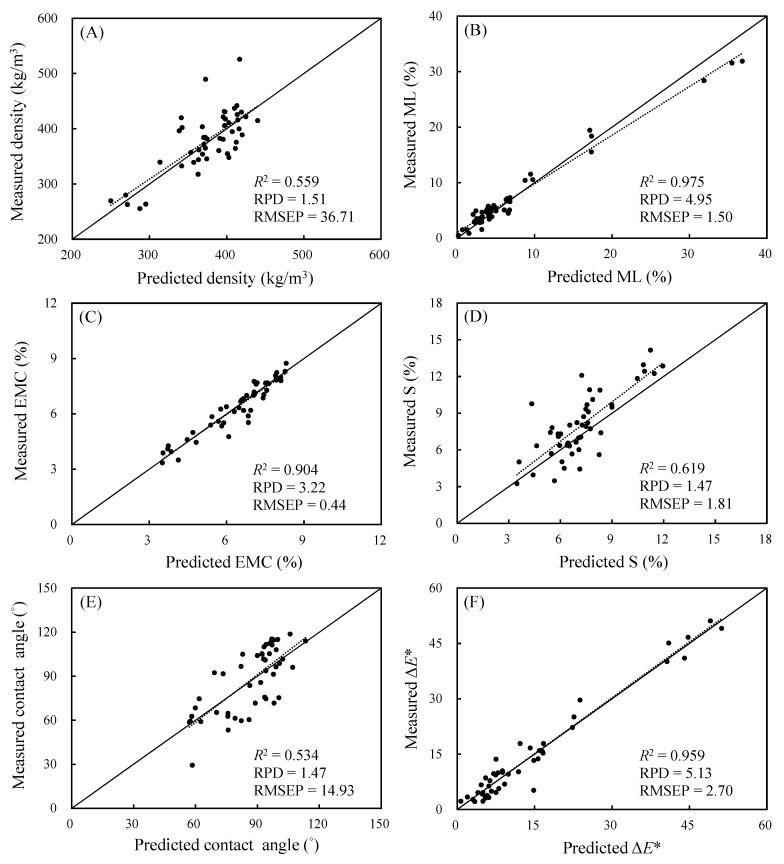
The correlation between predicted and measured values of air-dry density (**A**), ML (**B**), EMC (**C**), S (**D**), contact angle (**E**), and ΔE* (**F**) in prediction data.

To verify the accuracy of the prediction model for the aforementioned properties, the prediction sets in this model were substituted, and the *R*^2^ value, RMSEP, and RPD were calculated through comparison with the observed data to evaluate the model accuracy. Urbano-Cuadrado et al. [37] argued that the *R*^2^ value can indicate the dispersion of predicted and observed values in a model. A model with an *R*^2^ value of less than 0.3 is inadequate for quantification. A model with an *R*^2^ value between 0.3 and 0.5 can predict the high and low levels of properties. Furthermore, a model with an *R*^2^ value between 0.5 and 0.7 can predict the high, medium, and low levels of properties, and that with an *R*^2^ value greater than 0.7 can be used to conduct quantitative analysis. Additionally, Hsu et al. [38] indicated that the RPD is the standard deviation of observed values of the prediction set divided by the RMSEP. The value can be used to evaluate model accuracy; higher values indicate greater model prediction accuracy, with values greater than 2.5 generally acceptable. As presented in Figure 6, the *R*^2^ values of ML (0.975), EMC (0.904), and ΔE* (0.959) were greater than 0.7, and the RPDs for ML (4.95), EMC (3.22), and ΔE* (5.13) were greater than 2.5, indicating the high intensity of these data of the three properties and suggesting favorable model prediction performance and accuracy. The RPD for air-dry density was 1.51, indicating that this model can be used for screening (1.5 < RPD < 2.5). The *R*^2^ values of the S and contact angle ranged from 0.5 to 0.7, and their RPDs ranged from 1 to 1.5, indicating that the prediction models for these two properties can differentiate the high, medium, and low levels of these properties; the regression lines of the predicted and measured values were similar to the 45-degree line, indicating that they can be used for preliminary screening [39,40]. In sum, the aforementioned findings revealed that NIRS can be applied to accurately predict the ML, EMC, and ΔE* values of VH-treated luanta fir wood.

**Figure 7:**
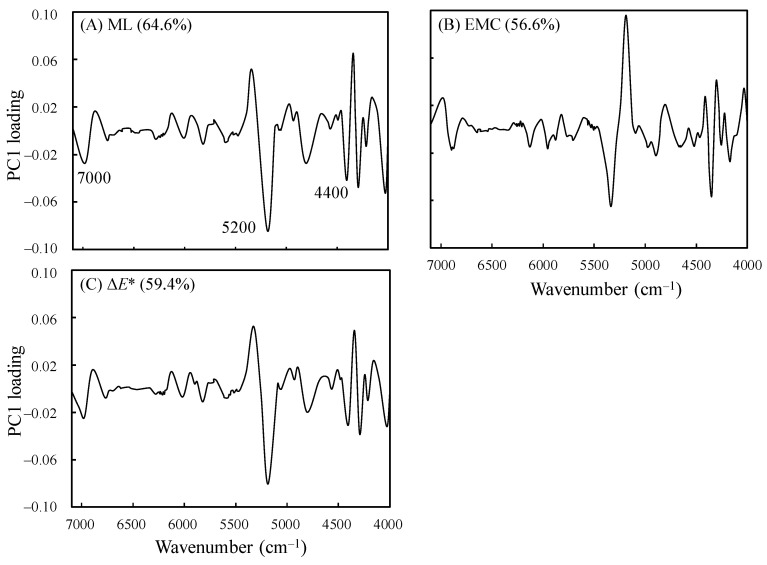
PC1 variable loading of the NIRS-based prediction model for ML (**A**), EMC (**B**), and ΔE* (**C**).

To identify the main influencing factors for the prediction model with RPDs of greater than 2.5 for ML, EMC, and ΔE*, a test was performed to further explore the PC1 variable loading of the NIRS-based prediction model. The analysis results are presented in Figure 7. As shown in Figure 7A,C, ML and ΔE* had similar influencing factors, including the characteristic absorption peak of the hydroxyl groups of the noncrystalline regions of cellulose at 7000 cm^−1^, the characteristic absorption peak of the hydroxyl groups in water molecules at 5200 cm^−1^, and the cellulose and hemicellulose C–H vibration absorption peak at 4400 cm^−1^. These factors indicated that the ML and ΔE* values increased when degradation occurred in the heated hydroxyl groups in the noncrystalline regions of hemicellulose and cellulose, and the moisture content in the wood decreased. By contrast, the influencing factors for EMC were opposite to those for ML and ΔE*. The EMC in the wood increased under the increased intensity of the three aforementioned characteristic absorption peaks, indicating that when the relative content of the hydroxyl groups, cellulose, and hemicellulose in the wood decreased, the moisture content decreased, and the ML and color difference increased. The findings also verified that alteration of the chemical properties of wood affects its physical properties.

## 4. Conclusions

In this study, luanta fir wood was modified through VH treatments with various conditions (160–240 °C and 4–16 h). In addition to exploring the physical and chemical properties of the wood, NIRS was used to create prediction models for the relevant properties. The test results demonstrated that the physical properties of VH-treated wood, including the ML, MEE, ASE, color difference, and surface contact angle, increased under increased treatment temperature and time. By contrast, the air-dry density of the wood decreased under increased treatment temperature and time. Moreover, the NIRS results revealed that, in the VH-treated luanta fir wood, thermal degradation occurred in the noncrystalline regions of hemicellulose and cellulose, and deacetylation and dehydration reactions were generated in hemicellulose, thereby decreasing the content of the hydroxyl and acetyl groups, and increasing the luanta fir wood’s crystallinity and dimensional stability. Additionally, degradation began to occur in the crystalline regions of cellulose under VH treatment at 240 °C for 8 h. Through using NIRS to create the prediction model for the physical properties of the VH-treated luanta fir wood, this study verified that this model is useful for air-dry density screening (1.5 < RPD < 2.5). In contrast, the RPDs of this prediction model for ML, EMC, and ΔE* were 4.95, 3.22, and 5.13, respectively, indicating the model was good with high prediction accuracy (RPD > 2.5). To the best of our knowledge, this is the first study to use this nondestructive technique to accurately predict the physical properties of VH-treated wood.

## Figures and Tables

**Table 1:** Mass loss (ML), air-dry density, equilibrium moisture content (EMC), moisture excluding efficiency (MEE), volumetric swelling coefficient (S), anti-swelling efficiency (ASE), and contact angle of luanta fir wood after VH treatment.

Sample	ML (%)	Air-Dry Density (kg/m^3^)	EMC (%)	MEE (%)	S (%)	ASE (%)	Contact Angle (°)
Control	-	416 ± 53 ^a^	8.3 ± 0.3 ^a^	-	13 ± 2 ^a^	-	60 ± 9 ^e^
160 °C/4 h	1.7 ± 1.4 ^i^	405 ± 47 ^ab,ns^	7.9 ± 0.4 ^ab,*^	5 ± 4 ^i^	12 ± 2 ^ab,*^	12 ± 16 ^e^	60 ± 14 ^e,ns^
160 °C/8 h	2.8 ± 0.7 ^ghi^	394 ± 41 ^ab,ns^	7.8 ± 0.4 ^ab,**^	6 ± 5 ^i^	9 ± 1 ^bc,***^	30 ± 6 ^de^	74 ± 21 ^bcde,*^
160 °C/16 h	3.9 ± 0.7 ^fgh^	374 ± 34 ^ab,*^	7.3 ± 0.4 ^bcd,***^	12 ± 5 ^ghi^	8 ± 1 ^cdef,***^	38 ± 10 ^bcd^	77 ± 15 ^abcde,*^
180 °C/4 h	2.4 ± 0.7 ^hi^	409 ± 36 ^ab,ns^	7.9 ± 0.6 ^ab,*^	5 ± 7 ^i^	8 ± 1 ^cde,***^	37 ± 9 ^bcd^	71 ± 14 ^de,*^
180 °C/8 h	3.3 ± 0.6 ^fghi^	389 ± 42 ^ab,ns^	7.4 ± 0.4 ^b,***^	11 ± 5 ^i^	7 ± 1 ^cdefg,***^	48 ± 8 ^abcd^	77 ± 12 ^abcde,**^
180 °C/16 h	4.7 ± 0.8 ^ef^	354 ± 37 ^abc,**^	6.5 ± 0.7 ^de,***^	22 ± 9 ^fg^	7 ± 1 ^cdefg,***^	45 ± 10 ^abcd^	97 ± 13 ^abcd,***^
200 °C/4 h	4.3 ± 1.3 ^fg^	389 ± 49 ^ab,ns^	7.4 ± 0.4 ^bc,***^	12 ± 5 ^hi^	9 ± 2 ^bcd,***^	33 ± 19 ^cde^	72 ± 23 ^cde,ns^
200 °C/8 h	4.7 ± 0.6 ^ef^	394 ± 56 ^ab,ns^	6.5 ± 0.2 ^def,***^	22 ± 3 ^ef^	7 ± 1 ^cdefg,***^	49 ± 7 ^abcd^	95 ± 12 ^abcd,***^
200 °C/16 h	6.4 ± 0.5 ^de^	370 ± 52 ^ab,*^	5.6 ± 0.8 ^gh,***^	33 ± 9 ^cd^	6 ± 1 ^defg,***^	53 ± 10 ^abc^	104 ± 12 ^a,***^
220 °C/4 h	4.9 ± 1.1 ^def^	414 ± 81 ^a,ns^	6.6 ± 0.4 ^cde,***^	21 ± 5 ^fgh^	7 ± 1 ^cdefg,***^	47 ± 9 ^abcd^	91 ± 13 ^abcd,***^
220 °C/8 h	6.6 ± 0.8 ^d^	367 ± 52 ^ab,*^	5.7 ± 0.3 ^fg,***^	32 ± 4 ^de^	6 ± 2 ^efg,***^	56 ± 12 ^ab^	99 ± 18 ^abc,***^
220 °C/16 h	9.6 ± 1.4 ^c^	358 ± 47 ^abc,*^	4.8 ± 0.2 ^hi,***^	43 ± 3 ^bc^	5 ± 1 ^fg,***^	59 ± 11 ^a^	100 ± 22 ^ab,***^
240 °C/4 h	6.1 ± 1.1 ^de^	367 ± 37 ^ab,*^	5.9 ± 0.5 ^efg,***^	30 ± 6 ^def^	7 ± 2 ^cdefg,***^	43 ± 12 ^abcd^	99 ± 17 ^abc,***^
240 °C/8 h	18.0 ± 1.3 ^b^	322 ± 41 ^bc,***^	4.1 ± 0.2 ^ij,***^	51 ± 3 ^ab^	5 ± 2 ^g,***^	63 ± 13 ^a^	97 ± 10 ^abcd,***^
240 °C/16 h	31.2 ± 1.2 ^a^	275 ± 32 ^c,***^	3.7 ± 0.3 ^j,***^	56 ± 4 ^a^	7 ± 2 ^cdefg,***^	45 ± 14 ^abcd^	96 ± 8 ^abcd,***^

Values are mean ± SD (*n* = 15). Column with different letters indicate significant differences (*p* < 0.05). ns: non-significant; *: *p* < 0.05; **: *p* < 0.01; ***: *p* < 0.001 (one-tailed test) compared with control.

**Table 2:** The ranges of physical properties of calibration and prediction sets in NIRS analysis model.

Properties	Calibration Data				Prediction Data			
	Min	Max	Mean	N	Min	Max	Mean	N
Air-dry density (kg/m^3^)	223	579	374	192	255	526	379	48
ML (%)	0.3	34.0	7.2	180	0.5	31.9	7.4	45
EMC (%)	0.9	13.2	8.3	192	1.2	12.9	7.9	48
S (%)	2.9	18.6	7.7	192	3.2	14.2	8.0	48
Contact angle (°)	29	121	85	192	53	119	86	48
ΔE*	2	54	15	180	2	51	15	45

N: number of sample data.

**Table 3:** Results of PLSR model by different preprocessing methods.

Properties	Preprocessing Method	LVs	*R* ^2^	RMSEC
Air-dry density	Original spectra	10	0.394	46.57
	MSC	10	0.386	46.88
	SNV	10	0.408	46.02
	MSC + baseline correction	10	0.395	46.52
	1stDer	10	0.755	31.13
	1stDer + SG	10	0.800	26.77
	2ndDer	10	0.892	20.81
	2ndDer + SG	10	0.935	15.07
ML	Original spectra	10	0.943	1.80
	MSC	10	0.963	1.45
	SNV	10	0.964	1.43
	MSC + baseline correction	10	0.963	1.45
	1stDer	10	0.986	0.90
	1stDer + SG	10	0.982	1.01
	2ndDer	10	0.959	0.26
	2ndDer + SG	10	0.998	0.38
EMC	Original spectra	10	0.879	0.51
	MSC	10	0.888	0.50
	SNV	10	0.893	0.48
	MSC + baseline correction	10	0.887	0.50
	1stDer	10	0.948	0.34
	1stDer + SG	10	0.936	0.37
	2ndDer	10	0.939	0.37
	2ndDer + SG	10	0.988	0.16
S	Original spectra	10	0.621	1.64
	MSC	10	0.598	1.69
	SNV	10	0.626	1.63
	MSC + baseline correction	10	0.604	1.67
	1stDer	10	0.872	0.95
	1stDer + SG	10	0.811	1.16
	2ndDer	10	0.805	1.17
	2ndDer + SG	10	0.952	0.55
Contact angle	Original spectra	10	0.513	14.68
	MSC	10	0.481	15.14
	SNV	10	0.521	14.56
	MSC + baseline correction	10	0.487	15.06
	1stDer	10	0.793	9.58
	1stDer + SG	10	0.715	11.23
	2ndDer	10	0.715	11.22
	2ndDer + SG	10	0.943	5.02
ΔE*	Original spectra	10	0.948	3.02
	MSC	10	0.952	2.88
	SNV	10	0.958	2.69
	MSC + baseline correction	10	0.952	2.88
	1stDer	10	0.979	1.91
	1stDer + SG	10	0.972	2.22
	2ndDer	10	0.948	0.56
	2ndDer + SG	10	0.996	0.83

**Table 4:** Results of PLSR model by different LVs.

Properties	LVs	*R* ^2^	RMSEC	RMSECV	Difference (%)
Air-dry density	1	0.220	50.96	51.18	0
	2	0.295	48.71	49.15	1
	3	0.377	46.09	51.55	12
	4	0.395	45.87	51.54	12
	5	0.522	40.84	52.80	29
	6	0.588	39.20	51.72	32
	7	0.713	33.75	52.19	55
	8	0.823	25.69	51.05	99
	9	0.883	20.82	51.30	146
	10	0.935	15.94	51.09	221
ML	1	0.781	3.56	3.58	1
	2	0.949	1.75	1.76	0
	3	0.954	1.70	1.73	2
	4	0.963	1.57	1.63	4
	5	0.967	1.48	1.66	13
	6	0.981	1.16	1.55	34
	7	0.988	0.90	1.48	65
	8	0.992	0.70	1.39	99
	9	0.996	0.54	1.37	155
	10	0.998	0.39	1.43	265
EMC	1	0.529	0.99	1.00	1
	2	0.848	0.57	0.57	1
	3	0.858	0.55	0.56	2
	4	0.870	0.53	0.55	3
	5	0.905	0.46	0.52	15
	6	0.927	0.40	0.52	29
	7	0.951	0.34	0.55	65
	8	0.964	0.30	0.54	83
	9	0.977	0.23	0.54	138
	10	0.988	0.17	0.54	220
S	1	0.297	2.08	2.10	1
	2	0.489	1.78	1.80	1
	3	0.510	1.75	1.78	2
	4	0.570	1.66	1.74	5
	5	0.676	1.44	1.67	16
	6	0.730	1.33	1.66	25
	7	0.819	1.13	1.68	49
	8	0.869	0.93	1.72	84
	9	0.927	0.70	1.72	147
	10	0.952	0.57	1.71	203
Contact angle	1	0.176	18.61	18.74	1
	2	0.372	16.36	16.53	1
	3	0.419	15.80	16.16	2
	4	0.446	15.46	15.90	3
	5	0.578	13.77	16.79	22
	6	0.673	12.02	16.43	37
	7	0.742	10.73	16.43	53
	8	0.833	8.70	16.40	88
	9	0.912	6.33	16.86	166
	10	0.943	5.13	16.91	230
ΔE*	1	0.657	7.70	7.76	1
	2	0.926	3.63	3.66	1
	3	0.937	3.40	3.59	6
	4	0.942	3.30	3.45	4
	5	0.962	2.66	3.30	24
	6	0.972	2.31	2.94	27
	7	0.981	1.92	2.90	51
	8	0.986	1.61	2.88	79
	9	0.991	1.31	2.80	114
	10	0.996	0.85	2.75	222

## Data Availability

The data presented in this study are available on request from the corresponding author.
